# Telehealth during and beyond the COVID-19 Pandemic: Evidence from licensed dietitians in an emerging economy

**DOI:** 10.1371/journal.pone.0311330

**Published:** 2026-02-06

**Authors:** Maya Assaad, Nour Chamma, Miroslav Mateev, Rana Rizk

**Affiliations:** 1 Abu Dhabi School, Abu Dhabi School of Management, Abu Dhabi, United Arab Emirates; 2 Department of Nutrition and Food Science, School of Arts and Sciences, Lebanese American University, Byblos, Lebanon; 3 INSPECT-LB (Institut National de Santé Publique, d’Épidémiologie Clinique et de Toxicologie-Liban), Beirut, Lebanon; Manipal Academy of Higher Education, INDIA

## Abstract

**Background:**

The sudden onset of the SARS-Cov-2 (COVID-19) pandemic disrupted access to in-person nutrition consultation and prompted the rapid adoption of telehealth by dietitians.

**Objective:**

This study investigates the use of telehealth among Lebanese Licensed Dietitians (LDs) during COVID-19, in the absence of national telehealth practical guidelines (TPG), and offers insights into its application amid overlapping crises including a pandemic, economic crisis, and infrastructure disruption in Lebanon.

**Design:**

A cross-sectional study conducted in March 2023, using an anonymous 44-question online survey, distributed via the Lebanese Order of Dietitians and social media platforms. Participants: Ninety-four dietitians participated (98.9% female, mean(SD) age: 30.54(6.41) years); mean(SD) experience: 7.89(5.7) years). Most reported practicing clinical nutrition as their primary practice area (87.2%), primarily in weight management (84%). Main outcome: Measures included Dietitians’ experience with telehealth, tools used in remote consultations, perceived barriers and facilitators, and perspectives on future application. Statistical analyses: Descriptive analysis (counts, frequencies) were analyzed using SPSS version 28.

**Results:**

Telehealth use rose from 48.4% before COVID-19 to 97.8% during it. Commonly used platforms included WhatsApp (90.3%), Zoom (72.0%), and e-mails (41.9%). Reported barriers included bad internet connection (74.2%), patients preferring face-to-face consultation (61.3%), and patients unfamiliar with emerging videoconferencing technologies (33.3%). Benefits included scheduling and time flexibility (83.9%), decrease in practice-related costs (77.4%), and compliance with social distancing measures (53.8%). Most respondents acknowledged that Telehealth is needed (78.5%) and applicable in the Lebanese context (64.6%) and called for telehealth trainings (78.5%) and national TPG development (74.2%).

**Conclusion:**

This study recognizes the growing use of telehealth in Lebanon, underscoring the need for telehealth with national regulations and evidence-based guidelines. Despite limited infrastructure, LDs continued delivering care, emphasizing the urgency for secure and standardized frameworks to support ethical and sustainable digital health practice.

## Introduction

The SARS-Cov-2 pandemic (COVID-19) sudden onset caused severe lifestyle changes impacting negatively on the mental and physical health of individuals. Moreover, it severely disrupted the access to hospitals and clinics for face-to-face consultation and therapy. To overcome these challenges, a paradigm shift toward digital health and remote clinical services was seen among healthcare providers and dietitians [1]. Facilitated by the emergence of information and communication technology (ICT) and direct-to-consumer virtual devices for data collection, telehealth became an alternative and effective tool for nutrition care in time of pandemic [2–4].

The public health emergency declaration of COVID-19 by the World Health Organization (WHO) followed by the lockdown imposed in March 2020 in Lebanon, have similarly exposed the medical sector to remote consultations, which became the “significant mediator for the resilience of the care system” [5]. Prior to COVID-19, the country was in the primary phase of incorporating telehealth practices into its health system with some hospital-based virtual services performed exclusively in the private sector [6]. Unfortunately, the legal infrastructure supporting the development of telehealth systems was very weak. The Law Decision No. 1/227 governing the introduction of electronic and digital services in Lebanon issued in 2013 was not implemented and official Telehealth practice guidelines (TPG) and standards were still missing [7]. The national E-Health Program foreseeing the incorporation of telehealth and mobile health in respect to the principles of e-Health ethics (confidentiality, credibility, and privacy of health information) was not yet fully integrated into the health system [7].

However, TPG and national policies were found to be essential for the proper implementation and assessment of remote services and for defining telehealth quality metrics and performance measurement [8]. As such, and in low-to-middle-income countries like Lebanon, telehealth experience during COVID-19 appeared to be one of a kind. In Lebanon, the pandemic event coincided with the occurrence of a severe economic crisis and a massive explosion at Beirut port. Consequently, the health system and the technological infrastructure in the capital were severely destroyed and hit simultaneously by liquidity crisis, bank withdrawal restrictions, lack of electricity, shortage in fuel, scarcity of medical equipment, hyperinflation and job losses [9]. Under these circumstances, the financial crisis and the lack of available resources constituted additional barriers facing the adoption of advanced technology and the maturation and proper implementation of telehealth [10]. In addition, barriers associated with socioeconomic status appeared to aggravate furthermore telehealth inequities and disparities in the access to care during the pandemic [11].

In this context, as with other healthcare providers, dietitians were increasingly engaged in remote practices. Those working in the field of clinical nutrition, which involves the provision of medical nutrition care for individuals with acute or chronic disease conditions, delivered in hospital or community-based settings, adapted to delivering services remotely [12]. In addition to the decrease in immunity and the exacerbation of chronic diseases and malnutrition reported elsewhere, the food shortages and rising food insecurity caused by the Lebanon’s socio-economic crisis placed the population at even a greater risk of malnutrition, further aggravating the health impact of COVID-19 [13].

To date, studies conducted on telehealth use among dietitians in the Arab world during the pandemic have focused on the change in the workload, caseload, tasks and responsibilities of hospital dietitians, and the challenges faced in the nutrition management of COVID-19 patients [14]. The use of social/mass media by dietitians to perform telehealth during COVID-19 was explored in Arab countries (such as in the UAE, KSA, Jordan…) and in Lebanon [15]. Telehealth uptake by Lebanese physicians and telehealth application for mental health amid the pandemic were studied, and findings on the increased use of telehealth via video consultations for diagnosis and treatment have been reported [6–17]. Even though COVID-19 has imposed the rapid adoption of telehealth, the performance of healthcare providers during telehealth sessions was notably enhanced in countries such as the UAE and KSA by the existence of legal frameworks and technological infrastructures supporting the effective implementation and delivery of remote services [18].

Thus, and since the Lebanese laws failed to determine in which practice telehealth can be eligible and applicable, in the absence of TPG, Lebanese physicians were urged to apply the general provisions related to their responsibility, liability, in-clinic medical errors and misdiagnosis to the virtual setting to properly conduct telehealth services [7].

Based on these observations, we may conclude that, although COVID-19 had caused a global surge in telehealth uptake, there is a deep gap in the literature concerning telehealth use in the absence of TPG, and in countries experiencing an economic crisis and underdeveloped infrastructures. On the other hand, telehealth modalities adopted by healthcare providers, and specifically by dietitians in the MENA region, to respond to the dynamic change induced by COVID-19 in the healthcare system are not well explored. Further, there is limited evidence on the importance of telehealth services for nutrition care in the post-COVID-19 period, and for the health care system stability in this region.

This study aimed to address this gap by conducting a cross-sectional online survey administered to Lebanese Licensed Dietitians (LDs) in March 2023 to examine telehealth uptake during COVID-19 and its sustainability beyond the pandemic. The survey explored telehealth modalities, challenges, benefits, and perspectives in the absence of TPG and amid ongoing infrastructure and economic challenges.

To achieve its aim, this study addresses the following research questions:

(1) Did the use of telehealth by Lebanese Licensed Dietitians (LDs) change at the onset of the COVID-19 pandemic?
(2) What tools and modalities did LDs use during telehealth consultations in the absence of Telehealth Practice Guidelines (TPG)?
(3) What were the main benefits and barriers to telehealth use among LDs in Lebanon?
(4) What were LDs’ perspectives on their telehealth experience during and after the pandemic?

This study contributes to understanding telehealth among LDs by analyzing its use during COVID-19 pandemic in the absence of national guidelines. The main contributions of this study are: (1) analyzing the use of telehealth among LDs amid the pandemic and in the absence of TPG, including the identification of perspectives, barriers, facilitators and benefits, (2) evaluating its applicability for nutrition care in the Lebanese context, (3) identifying challenges facing the continuous improvement of telehealth and its future implications in Lebanon, (4) proposing state-of-the-art recommendations for telehealth sustainability beyond COVID-19 pandemic, including the integration of authorized telehealth platforms into the Lebanese health system and the development of national policies, technical regulations, standards, protocol of care, and professional development, and (5) promoting telehealth as a strategic and sustainable model to be integrated into routine dietetic practice in countries affected by conflict and economic instability.

## Materials and methods

### Survey development and design

A cross-sectional study was conducted using an online questionnaire adapted from a previously published, peer-reviewed questionnaire [19]. The original questionnaire was modified by removing questions that were not relevant to Lebanese context (such as questions regarding the estimated percentage of the patient population covered by Medicare). The study questionnaire included 44 questions divided into four sections covering LDs’ demographic, educational and professional characteristics; telehealth experience before and during the pandemic; tools used; barriers, facilitators and benefits; and LDs’ perspectives on their telehealth experience. The questionnaire was pilot-tested, and feedback from the pilot was used to finalize its design. The inclusion criteria were: Lebanese dietitian, holding a bachelor’s degree in human nutrition and dietetics, licensed by the Lebanese Ministry of Public Health (MOPH), and practicing in Lebanon during COVID-19 pandemic.

### Data collection

Due to the absence of complete list of LDs in Lebanon, and to maximize survey outreach and minimize the risk of undelivered emails, a link to the Google Forms survey was distributed via the Lebanese Order of Dietitians, social media platforms (WhatsApp, LinkedIn, and Facebook), as well as through nutrition faculties and alumni networks at Lebanese universities. To prevent overlapping and duplication in data collection, LDs who had already completed the questionnaire were asked not to participate. Data were collected using a self-administered, anonymous Google Forms questionnaire. The survey was designed to ensure participant confidentiality, with no personally identifiable information collected. Access to the responses was restricted to the research team. Participation was voluntary, and respondents provided informed consent at the beginning of the survey.

### Ethical considerations

The study was reviewed and approved by the Lebanese American University Institutional Review Board (LAU IRB) under the reference LAU.SAS.RR1.29/Nov/2022. Informed consent was implied through voluntary completion of the questionnaire. Google Forms settings were configured to ensure that personal data, such as emails and IP addresses, were not collected. The participation in the study was anonymous, voluntary, and non-payable.

### Statistical analysis

The survey data were managed and analyzed descriptively using Statistical Package for the Social Sciences (SPSS), Version 28. Means and standard deviations (SD and median (IQR) were reported for continuous variables, while counts and percentages were used for categorical variables.

### Inclusivity in global research

Additional information regarding the ethical, cultural, and scientific considerations specific to inclusivity in global research is included in the Supporting Information (S3 File Inclusivity in Global Research Questionnaire).

## Results and discussion

### Participants’ characteristics

In total, 94 LDs were included in this study. The sample consisted predominantly of females (98.9%) and young dietitians (mean (SD) age: 30.54 (6.41) years), with a master’s degree (58.5%), and a median (IQR) of 6.00 (3.00, 12.00) years of experience in dietetics practice. Most respondents (87.2%) identified clinical nutrition as their primary practice area, with a focus on weight management (84%), food and nutrition consultation (58.5%), and diabetes care (45.7%). S1 Table describes the socio-demographic characteristics of the participants.

### Telehealth experience during COVID-19 pandemic

Although 48.4% (N = 45) of respondents reported using telehealth for nutrition care before the pandemic; this proportion increased significantly to 97.8% (N = 91) during COVID-19 pandemic. LDs who had prior experience with telehealth before the onset of the pandemic reported a mean (SD) of 1.43 (2.5) years of use. During the pandemic, and as illustrated in S1 Fig, the main tools used for delivering telehealth nutrition care were WhatsApp (90.3%), Zoom (72.0%), E-mails (41.9%), and telephone (39.8%). During telehealth sessions, LDs relied primarily on video calls (83.9%), followed by voice calls (67.7%) and voice messages (65.6%).

Most respondents (51.6%) spent more than 30 minutes in direct contact with individuals or groups to collect information on food and nutrition-related history (94.6%), patient history (89.2%), patient behavior (74.2%) and, knowledge, beliefs, and attitudes (72.0%). LDs reported spending an average of 6.4 hours per week of face-to-face counseling prior to the pandemic. While this may not reflect their total clinical workload, session lengths and patient volume vary by setting, where brief consultations were common during hospital rounds and longer sessions occurred in outpatient or private clinics settings. Moreover, some LDs worked part-time or saw patients based on availability, particularly in private practice. During the pandemic, the reported 30 minutes per telehealth session refers to the duration of individual remote consultations and reflects the additional time that LDs might need to collect comprehensive dietary histories and patient information in a virtual setting. The results of telehealth sessions were most frequently communicated to the referring medical providers through social media applications (40.9%), e-mail (21.5%), and electronic medical record (20.4%). The experience of LDs with telehealth for nutrition care before and during the pandemic is summarized in S2 Table.

The types of nutrition assessment and monitoring conducted via telehealth during the pandemic are illustrated in S3 Fig.

During the COVID-19 pandemic, the most reported activities among LDs included the collection of food and nutrition-related history (94.6%) followed by patient history (89.2%), assessment of physical activity, behavior, beliefs and attitudes, as well as evaluation of biochemical data, anthropometrics, medication use, food access barriers, and nutrient administration. S3 Fig displays the types of nutrition assessments and monitoring, or evaluation activities conducted remotely by Lebanese Licensed Dietitians (LDs).

### Barriers, facilitators, benefits and perspectives related to telehealth use

The reported barriers, facilitators and benefits of telehealth use during COVID-19 pandemic are figured in S3 Table. The most frequently reported barriers to providing telehealth nutrition care were bad connections (74.2%) followed by patients preferring face-to-face consultation (61.3%), and patients lacking technical literacy (33.3%). Among the benefits, LDs cited scheduling flexibility (83.9%), reduced practice-related costs (77.4%), the promotion of technology acceptance and penetration (69.9%), and compliance with COVID-19 social distancing measures (53.8%). At this point, it is worth noting that the reported 6.4 hours per week reflects specifically the one-on-one counseling, whereas the 10.7 hours indicated in S3 Table represents the total weekly time dedicated to face-to-face nutrition care, including counseling, inpatient visits, group sessions, and educational activities. Unfortunately, and as shown in S2 Fig, during the pandemic, most respondents did not receive any training on how to properly conduct telehealth (93.5%) and did not consult any specific guidance (94.6%). Moreover, participants rated their overall experience with telehealth during the pandemic at a mean (SD) of 6.62 on a scale from 1 to 10. A majority agreed that telehealth is needed in Lebanon (78.5%), 64.6% believed it is applicable in the Lebanese context, and a 81.7% expressed their willingness to continue using telehealth after the pandemic. Additionally, 78.5% indicated interest in receiving future telehealth training for nutrition care, and 74.2% reported the need for specific TPG to ensure proper delivery of remote services (74.2%).

This study is the first to investigate telehealth use among LDs prior and during COVID-19 pandemic in the absence of national Telehealth practice guidelines (TPG), standards and best practices to ensure ethical and safe practices. It identifies telehealth tools used, as well as the perceived benefits and barriers, and offers insights into the applicability, effectiveness, and future sustainability of telehealth in the Lebanese context. Amid COVID-19 pandemic, this study reports an increase in telehealth use among LDs, reflecting trends observed among other healthcare professionals in Lebanon, and dietitians in other countries [6–20]. Remarkably, most LDs (97.8%) reported using telehealth for nutrition care amid COVID-19 pandemic. In time of pandemic, telehealth has been perceived as a viable tool able to overpass lockdown measures, ensure patient safety, maintain the cycle of life, and support mental health and quality of life [12, 14-19]. Interestingly, we note a high use of telehealth practices among LDs before COVID-19 pandemic (48.4%), compared to only 15% of dietitians in Italy and 37% of dietitians in the US who performed telehealth activities prior the pandemic [12]. This early adoption could be related to the fact that some telehealth activities were initiated in the country before the pandemic onset as alternative practices to encompass the impact of the socioeconomic crisis, notably the scarcity of resources, lack of job opportunities, and limitation of transportation [6–21].

Given the limited financial resources, LDs might have resorted to remote modalities for delivering nutrition care. However, despite some pre-pandemic telehealth activities, the majority still focused on face-to-face consultations in clinics (82%) and hospitals, suggesting that pre-pandemic telehealth practices were primarily used for follow-up with existing patients and/or for continuous education. Similar pre-pandemic patterns were noted among Lebanese physicians who, with limited financial resources caused by the economic crisis, were unable to travel to attend international conference and relied mainly on telehealth for continuing medical education. [6] Additionally, the use of videoconferencing for telehealth consultations was reported to be low among Lebanese physicians before the pandemic [6]. In contrast, the COVID-19 pandemic imposed a rapid shift toward audiovisuals tools and videoconferencing. LDs embraced telehealth, with their uptake being centered in private practices and among newly graduated LDs. In Lebanon, private practice is a common but self-managed form of employment, often represented under “clinics” in our data. This swift telehealth uptake observed in the country could be explained by the fact that LDs population was relatively young (mean (SD) age: 30.54 (6.41) years), already familiar with new technologies and accustomed to using smart applications, which came in similar to a study conducted on Italian dietitians.^20^ Comparable findings were also observed in the MENA region [14]. Thus, LDs initiated telehealth amid COVID-19 pandemic by relying on WhatsApp (90.3%) as a main tool, followed by Zoom (72.0%), Emails (41.9%) and phone calls (39.8%). Similarly, Lebanese physicians reported using WhatsApp (79%), phone calls (77%) and email (63%), along with platforms such as Zoom and social media (e.g., Facebook, YouTube and Instagram) [6]. Remarkably, during the pandemic, the use of mobile applications/software as telehealth tools was highly observed in low-to-middle income countries, with a strong preference for audio-visuals tools such as WhatsApp, Zoom, Facebook Messenger and Skype [22]. This was reflected in our study, which showed that LDs were able to select telehealth tools suited to the country’s economic status, despite the absence of official guidance. These tools offered LDs an atmosphere similar to in-clinic consultations, enabling direct interactions with patients (both within and outside Lebanon), as well as data collection, feedback, and evaluation. In contrast, the use of Zoom for healthcare and electronic medical record was minimal (around 3%) and exclusively reported among LDs working in hospitals. In fact, Electronic Healthcare Records were incorporated in some major private hospitals before the pandemic, but their use was limited to the context of some practices [6]. Moreover, telehealth platforms available in Lebanon (such as TrakMD, SohatiDoc, Doctori, and Drapp) were not authorized by MOPH and the Lebanese Order of Physicians [23]. Due to these facts, both Lebanese physicians and patients reported a preference for using WhatsApp instead of telehealth platforms as it allowed for spontaneous, informal and free of charge communication [23]. The findings of this study reflect limited integration of telehealth into the Lebanese nutrition care system prior to the pandemic. which hindered LDs’ ability to leverage existing infrastructure and deliver remote services effectively. As a result, LDs were not professionally equipped to initiate remote consultations, and both their profession and resilience were challenged by the pandemic. This may explain their reliance on mobile and social media applications (such as WhatsApp, Facebook Messenger…) (40.9%) and direct emails (21.5%) to communicate with referring medical providers. Social media applications thus emerged as alternative telehealth tools, enabling direct communication with patients, medical providers and colleagues, as well as indirect communication with virtual profiles via its platforms. In contrast to other scientific-based applications and business communication platforms, social media provided a user-friendly environment that enabled LDs to create and disseminate dietetic content using graphics, videos and interactive tools which allowed broader engagement and interdisciplinary integration. As a result, dietitians were able to go beyond counselling and nutrition care to address concerns, social trends, and common interests experienced in times of pandemic [14]. In the same directions, Rozga et al. (2021) found that dietitians preferred social media due to its accessibility and ease of interaction compared to rigid institutional platforms [19]. Similarly, Bookari et al. (2023) reported that Arab dietitians relied on social media to connect with diverse audiences and found it effective in addressing public concerns and social trends during the pandemic [15]. Furthermore, Alboraie et al. (2022) pointed out the regulatory, technical and infrastructural challenges of formal telemedicine platforms, making social media a more feasible and accessible communication tool [24].

Building on these observations, this study acknowledges that in the absence of authorized telehealth systems and a legal framework, and with the presence of underdeveloped infrastructure, a socio-economic crisis and limited resources, resorting to social media during the pandemic supported the resilience of LDs and provided them with business opportunities and marketing experience for professional growth. In the same direction, the use of social media as a telehealth tool was widely reported in other Arab countries (such as the UAE, KSA and Jordan), and appeared to be an effective telehealth tool during COVID-19 pandemic, despite ongoing concerns about privacy and data misuse [14]. Recent studies conducted in these countries, including Lebanon, revealed a significant shift toward telehealth during the COVID-19 pandemic. More than 63% of dietitians reported high reliance on social and mass media platforms, with Instagram emerging as the most commonly tool used (51.7%) [15]. Key benefits of these platforms included broader outreach and quick information sharing, despite challenges related to time constraints and effective communication [15]. During remote nutrition care, LDs identified several barriers centered around bad connections (74.2%), patients preferring face-to-face consultation (61.3%) and patients lacking technical literacy (33.3%). They expressed discomfort during telehealth sessions and faced challenges specific to the dietetic profession, such as difficulties in collecting physical data and conducting comprehensive nutrition assessments (32.3%). In addition, cultural barriers related to privacy concerns were identified in our study, including patient discomfort with discussing personal health information in the virtual settings. These findings are consistent with reports from the United States and several Arab countries. For example, Bookari et al. (2022) highlighted how sociocultural norms in Arab countries, particularly those related to gender roles and concerns about confidentiality, affect telehealth communication, especially in shared household spaces [14]. [14–24]. Similarly, Egyptian healthcare providers observed patient resistance to telehealth consultations due to cultural expectations around trust, confidentiality, privacy and the absence of secure communication systems [24]. In the US, Rozga et al. (2021) also reported that dietitian encountered patient reluctance to engage in telehealth sessions due to lack of privacy in home settings [19]. The use of commercially available platforms such as WhatsApp and Zoom for telehealth raises significant concerns regarding patient privacy and data security. In Lebanon, the absence of enforceable data protection laws and telehealth-specific regulations aggravated these risks and underscores the need for legal frameworks and TPG to govern digital practices and protect patient confidentiality [7].

One of the notable findings in this study is that 50.5% of LDs reported delivering nutrition care to patients located outside Lebanon. Under normal circumstances, and due to professional licensing restriction, such cross-border practices could have legal implications. However, this became possible during the pandemic due to the temporary relaxation of telehealth regulations, including regulatory adjustments such as waived licensure requirements to enable broader telehealth services [19]. It is worth noting that these international consultations were provided independently by LDs on a private basis and, in the absence of formal regulations governing cross-border telehealth practices, were neither affiliated with nor reimbursed by any national health system. In similar, Lebanese physicians practiced telehealth during the COVID-19 pandemic in an unregulated environment and expressed the need for regulatory frameworks to guide and protect their remote activities [6]. This highlights a systemic gap in telehealth governance in Lebanon and a lack of knowledge regarding the legal and ethical aspects of telehealth use across countries and health professions, underscoring the need for official TPG and professional training. Similarly, only a minority of physicians in KSA reported being aware of existent regulations governing the proper delivery of telehealth via smart devices, further emphasizing the need for clear regulatory guidance [25]. Nevertheless, LDs recognized several telehealth benefits, including scheduling flexibility (83.9%), decrease in practice-related costs (77.4%), and improved compliance with social distancing measures (53.8%). The mean (SD) rating of their overall experience scored 6.62, on a scale from 1 to 10. LDs found telehealth to be effective and applicable in Lebanon and expressed a strong need for the development of TPG and related trainings. They were also aware of the absence of a legal framework and professional support to ensure protection of professional practice, confidentiality, security, and privacy (S3 Table). Similar needs were reported by dietitians in Italy and in the UAE [12,14].

In conclusion, the study findings align with those reported among dietitians in other countries and among medical professionals in Lebanon. The Lebanese telehealth experience closely mirrors that of the MENA region during COVID-19 pandemic [26]. Yet, we demonstrate that telehealth is effective, applicable and necessary in the Lebanese context, serving as a resilient alternative for delivering care in time of pandemic, disasters, and severe crisis, with strong willingness among LDs to continue its use beyond COVID-19 pandemic.

## Strengths and limitations

This study was a pioneer in exploring telehealth use among LDs in the absence of TPG during the COVID-19 pandemic. To provide meaningful results, enable comparisons, and increase the survey’s reach, we adapted a previously validated questionnaire to the Lebanese context, and diffused it via online survey through official channels and social media platforms. As such and in a short period of time, data were collected simultaneously from multiple participants, without influencing any of their responses. However, this study has several limitations. The results were based on self-reported data rather than observed behavior, and data collection might be subject to social desirability bias such as participants might have changed their answers to reflect more professional practices. Further, the survey was distributed in selected professional Facebook groups, introducing potential selection bias. Participants who have previously used telehealth might have been pre-selected by the topic, i.e., being subject to self-selection. Moreover, as noted in other studies, the LDs population was relatively young and had limited years of professional experience in dietetic practice [12, 14-19]. All these limitations might impact the generalizability of the findings to the broader LDs community. Finally, since the study was conducted during the later stages of the pandemic, some answers might have been subject to recall bias.

## Recommendations and future implications

Conducted in March 2023 during the final stages of the pandemic, this study explores telehealth use across multiple waves of the virus and lockdowns, within the context of Lebanon’s ongoing economic crisis. It supports the use of telehealth as a potential strategy for delivering nutrition care during crisis and in settings affected by economic instability and conflict [22,27]. More, the benefits reported in this study support the sustainability and future implications of telehealth beyond the pandemic in Lebanon. As demonstrated in the literature and reflected in our study, LDs who used telehealth prior to COVID-19 pandemic showed greater willingness to continue its use, underscoring their role in sustaining such practices [28]. Telehealth offers low-to-middle income countries like Lebanon an opportunity to advance the Sustainable Development Goals (SDGs) related to quality, accessibility, and availability of health services for all [29] However, telehealth sustainability requires secure, regulated platforms that protect patient data, uphold confidentiality and, and preserve the integrity of the dietitian-patient relationship [28]. While relaxed regulations during the pandemic permitted the use of social media and mobile applications, the adoption of reliable platforms with robust privacy safeguards is now essential [30]. Given Lebanon’s limited infrastructure, a unified national telehealth nutrition platform for both the public and private sectors is recommended. This platform should be aligned with tools such as the Academy of Nutrition and Dietetics Health Informatics Infrastructure (ANDHII) to ensure interoperability between different health systems [31,32]. Developing TPG for nutrition care is essential for establishing a safe, standardized and evidence-based practice. These key elements can be translated into recommendations for the newly elected Lebanese Order of Dietitians to collaborate with the Ministry of Public Health (MOPH) and other stakeholders in developing and harmonizing a legal framework for telehealth. This framework should include national policies, technical regulations, standards and protocol of care. To ensure equitable access, these regulations must be supported by the adoption of innovative technologies that promote telehealth accessibility for all citizens and residents. Beyond COVID-19 pandemic, sustaining telehealth requires system accreditation to ensure quality assurance, continuous improvement, performance evaluation, standardized protocols and operations, the promotion of a telehealth culture and acceptance, and the protection of patient experience [33]. Developing a skilled telehealth workforce is essential to overcome resistance to new technologies [25–30]. Accordingly, we recommend continuous training and professional development for LDs and other healthcare providers, on the proper and effective use of telehealth tools and modalities, as well as the ethical, legal, privacy, and cultural considerations that govern virtual care.

The exceptional situation experienced during the pandemic accelerated the integration of telehealth and reshaped the role of dietitians, catalyzing a paradigm shift in nutrition care delivery. LDs were introduced to innovative virtual care models and new modes of service delivery, highlighting the need to adapt to a rapidly evolving digital landscape. As a result, the sustainability of the dietitian’s career is now closely linked to their ability to embrace innovative practices and align their role with emerging technologies of the Fourth Industrial Revolution, including big data, genomics, and artificial intelligence [33]. Furthermore, personalized and precision nutrition care is expected to replace traditional treatment-oriented services, positioning telehealth platforms as a routine component of dietetic practice [2]. To support this transformation, innovation in telehealth and digital health for nutrition care will be essential in Lebanon in the near future.

## Conclusion

This study demonstrated a significant increase in telehealth use among LDs during the COVID-19 pandemic and reported overall satisfaction despite the absence of national regulations, TPG, and professional training. More, this study highlights the structural and institutional gaps limiting the effective integration of telehealth in LDs’ routine practices. In this context, the findings serve as a foundation upon which telehealth infrastructure and national TPG for nutrition care can be developed. By presenting the experience of Lebanon, it identifies telehealth as a strategic model applicable not only in time of pandemic and public health emergencies but also in time of economic crisis and resource scarcity.

However, telehealth sustainability remains dependent on policy development, professional development and technological adoption. Hence this study offers key insights for ensuring the future sustainability of telehealth and supporting the adoption of innovative, patient-centered technologies. Further research is needed in Lebanon to evaluate the long-term effectiveness of telehealth for nutrition care, its outcomes, the quality performance of its actors, and the patient experience.

## Supporting information

S1 File PLOS’ questionnaire on inclusivity in global research. Completed version of the PLOS Inclusivity in Global Research Questionnaire, detailing research context, participant engagement, and ethical considerations.
(PDF)

S1 Dataset Raw dataset in Microsoft Excel (xlsx) format. This file contains anonymized responses from 94 participants and includes all data analysed in the study.
(XLSX)

S1 Table Socio-Demographic Characteristics of a Cross-sectional Sample of Licensed Dietitians (LDs) Practicing in Lebanon and using Telehealth during the COVID-19 Pandemic (N = 94). This table summarizes the socio-demographic characteristics of 94 Licensed Dietitians (LDs) practicing in Lebanon who participated in an anonymous, cross-sectional online survey conducted in March 2023. Reported characteristics include age group, gender, region of residence, highest level of education, years of professional experience, primary area of practice, and employment sector. Percentages may not total 100% due to rounding or missing responses.
(DOCX)

S2 Table LDs Respondents’ Experience in Providing Telehealth for Nutrition Care Prior to and During COVID-19 in Lebanon (N = 93). Legend: This table summarizes the experience of 93 Licensed Dietitians (LDs) in Lebanon in providing nutrition care via telehealth before and during the COVID-19 pandemic. It includes data on telehealth adoption, frequency of use, session duration, communication platforms, types of services provided, and the geographic scope of care delivery.
(DOCX)

S3 Table LDs Reported Barriers, Facilitators and Benefits of Telehealth Use during COVID-19 in Lebanon (N = 93). This table outlines the perceptions of 93 Licensed Dietitians (LDs) in Lebanon regarding key factors influencing the use of telehealth during the COVID-19 pandemic. Reported items include technological challenges, professional and institutional support, patient engagement, and perceived advantages of telehealth for delivering nutrition care.
(DOCX)

S1 Fig Tools Used by LDs to Provide Telehealth Nutrition Care During the Pandemic (%) (N = 93). Legend: This figure illustrates the types of tools and platforms utilized by 93 Licensed Dietitians (LDs) in Lebanon to deliver nutrition care via telehealth during the COVID-19 pandemic. Reported tools include videoconferencing applications, phone calls, messaging apps, and social media platforms. WhatsApp (90.3%) and Zoom (72.0%) were the most used platforms, followed by email (41.9%) and regular telephone calls (39.8%). Respondents were allowed to select multiple options. Other tools included social media applications, Microsoft Teams, Skype, Zoom for Healthcare, and electronic health records. Percentage reflects the proportion of respondents who indicated using each tool.
(TIF)

S2 Fig Perspective of LDs on Telehealth Use for Nutrition Care during The Pandemic (N = 93). Legend: This figure presents the perceptions of 93 Licensed Dietitians (LDs) regarding telehealth use for nutrition care during the COVID-19 pandemic related to applicability, effectiveness, patient engagement, and professional confidence associated with telehealth delivery. On a Likert Scale, respondents rated their level of agreement with five statements addressing the need for practical guidelines, future training, continued telehealth use post-pandemic, the applicability of telehealth in the Lebanese Context, and the overall need for telehealth in Lebanon. Responses are shown across five categories: strongly disagree, disagree, neutral, agree, and strongly agree.
(TIF)

S3 Fig Types of nutrition assessment and/or monitoring and evaluation conducted via Telehealth during COVID-19 (%) (N = 93). Legend: This figure shows the types of nutrition assessment and monitoring, or evaluation methods performed by 93 Licensed Dietitians (LDs) during the COVID-19 pandemic. Categories include dietary recall, anthropometric tracking, clinical and biochemical data collection, and behavioral assessments. Percentages represent the share of respondents who reported conducting each assessment type remotely.
(TIF)
