# Supplementary material for: Telehealth during and beyond the COVID-19 Pandemic: Evidence from licensed dietitians in an emerging economy
Source: PLoS One. 2026 Feb 6;21(2):e0311330. doi: 10.1371/journal.pone.0311330 (PMC12880700; doi:10.1371/journal.pone.0311330)
Supplement: S1 Table — This table summarizes the socio-demographic characteristics of 94 Licensed Dietitians (LDs) practicing in Lebanon who participated in an anonymous, cross-sectional online survey conducted in March 2023. Reported characteristics include age group, gender, region of residence, highest level of education, years of professional experience, primary area of practice, and employment sector. Percentages may not total 100% due to rounding or missing responses. (DOCX) [file pone.0311330.s003.docx]

| **Table 1.** The Socio-Demographic Characteristics of a Cross-Sectional Sample of LDs Practicing in Lebanon and Using telehealth During COVID-19 (N=94) | |
| --- | --- |
| **Survey questions** | ***N (%)*** |
| **Highest degree earned** | |
| Bachelor’s | 33 (35.1) |
| Master | 55 (58.5) |
| Doctorate | 6 (6.4) |
| **Focus area in which most time is spent^b^** | |
| Renal nutrition | 18 (19.1) |
| Diabetes care | 43 (45.7) |
| Gerontological (Elderly) nutrition | 11 (11.7) |
| Weight management | 79 (84) |
| Eating disorders | 25 (26.6) |
| Oncology | 10 (10.6) |
| Pediatric nutrition | 24 (25.5) |
| Food and nutrition consultation^e^ | 55 (58.5) |
| Generalist^f^ | 14 (14.9) |
| Other^a^ | 52 (55.3) |
| **Life stages of populations worked with^a^** | |
| Adults (ages 22-64) | 94 (100) |
| Older adults (age 65+) | 49 (52.1) |
| Teenagers and young adults (ages 13-21) | 70 (74.5) |
| Children (ages 6-12) | 47 (50) |
| Pregnant/ postpartum women | 52 (55.3) |
| Young children (ages 1-5) | 21 (22.3) |
| Infants | 8 (8.5) |
| **Practice area in which most time is spent^a^** | |
| Clinical nutrition^g^ | 82 (87.2) |
| Community nutrition^h^ | 19 (20.2) |
| Consultancy^i^ | 25 (26.6) |
| Education^j^ | 34 (36.2) |
| Entrepreneurship^k^ | 11 (11.7) |
| **Practice area in which 20% of your time is spent^a^** |  |
| Clinic | 49 (52.1) |
| Hospital | 18 (19.1) |
| Primary care center | 8 (8.5) |
| Other^c^ | 62 (66) |
|  | ***Median (IQR))^d^*** |
| **Years of experience as LDS** | 6.0 (3.0, 12.0)) |
| **Hours per week providing face-to-face nutrition care prior to COVID-19** | 6 (2.5, 15.7) |
| **Average number of patients consulted per day** | 4 (3.0, 8.0) |

LDs, Lebanese Licensed Dietitians; COVID-19, SARS-Cov-2 pandemic

a Respondents were able to select all options that applied.

b Pregnancy nutrition, hypertension, dyslipidemia, sports nutrition, pediatric nutrition, children with disabilities, gastrointestinal diseases

c Academia, consultancy, public health nutrition, community nutrition, food service, food safety and quality control

d Median (IQR) = Interquartile range

^e^ Food and Nutrition Consultation = One-on-one or group sessions focused on dietary advice and nutritional planning.

^f^ Generalist = A dietitian who works across multiple nutrition domains without a primary area of specialization.

^g^ Clinical Nutrition = Nutrition care provided in hospitals or healthcare settings for disease management.

^h^ Community Nutrition = Nutrition programs or services targeted at groups within the community to promote health and prevent disease.

^i^ Consultancy = Independent or contract-based nutrition guidance provided to individuals, organizations, or institutions.

^j^ Education = Teaching or academic responsibilities, often in universities or training programs.

^k^ Entrepreneurship = Business-related nutrition services such as private practice, product development, or digital platforms.
