# Supplementary material for: Telehealth during and beyond the COVID-19 Pandemic: Evidence from licensed dietitians in an emerging economy
Source: PLoS One. 2026 Feb 6;21(2):e0311330. doi: 10.1371/journal.pone.0311330 (PMC12880700; doi:10.1371/journal.pone.0311330)
Supplement: S2 Table — Legend: This table summarizes the experience of 93 Licensed Dietitians (LDs) in Lebanon in providing nutrition care via telehealth before and during the COVID-19 pandemic. It includes data on telehealth adoption, frequency of use, session duration, communication platforms, types of services provided, and the geographic scope of care delivery. (DOCX) [file pone.0311330.s004.docx]

| **Table 2.** LDs Respondents’ Experience in Providing Telehealth for Nutrition Care Prior to and During COVID-19 in Lebanon (N=93)^⁕^ | |
| --- | --- |
| ***Survey Questions*** | ***N (%)*** |
| **Provided nutrition care via Telehealth prior to Covid-19** |  |
| Yes | 45 (48.4) |
| No | 48 (51.6) |
| **Received training on the use of Telehealth prior to Covid-19** |  |
| Yes | 8 (8.6) |
| No | 85 (91.4) |
|  | **Mean(SD^a^)** |
| **Years of experience in providing nutrition care via Telehealth prior to Covid-19** | 1.43 (2.5) |
| **Hours per week providing face-to-face nutrition care prior to COVID-19 pandemic** | 10.7 (11.5) |
|  | **N (%)** |
| **Provided nutrition care via Telehealth for patients in Lebanon during Covid-19** |  |
| Yes | 91 (97.8) |
| No | 2 (2.2) |
| **Provided nutrition care via Telehealth for patients outside Lebanon during COVID-19** | |
| Yes | 47 (50.5) |
| No | 28 (30.1) |
| **Provided nutrition care via Telehealth from Lebanon during COVID-19** | |
| Yes | 92 (98.9) |
| No | 1 (1.1) |

| **Targets of nutrition care via Telehealth during COVID-19** |  |
| --- | --- |
| Individuals | 70 (75.3) |
| Both individuals and groups | 23 (24.7) |
| **Telehealth features used during Covid-19**b | |
| Voice call | 63 (67.7) |
| Text message | 53 (57.0) |
| Voice message | 61 (65.6) |
| Media (photo, video, radiological imaging, laboratory results…) | 44 (47.3) |
| Video calls | 78 (83.9) |

| **Types of nutrition interventions^c^ provided via Telehealth during COVID-19**b | |
| --- | --- |
| Nutrition counseling | 84 (90.3) |
| Nutrition education | 82 (88.2) |
| Coordination of nutrition care with healthcare professionals | 26 (28.0) |
| Food and/ or nutrient delivery | 20 (21.5) |
| Enteral and parenteral nutrition | 6 (6.5) |
| Population-based nutrition action | 8 (8.6) |
| Other^d^ | 53 (57.0) |

| **Patients consulted via Telehealth during Covid-19** |  |
| --- | --- |
| Patients you already know | 83 (89.2) |
| Patients you already Know and new patients | 66 (71.0) |
| New patients | 76 (81.7) |
| **Method of communicating results of Telehealth back to referring medical provider during COVID-19**b |  |
| I documented the interaction through an electronic medical record | 19 (20.4) |
| I sent an e-mail to the medical provider | 20 (21.5) |
| I sent a fax to the medical provider | 1 (1.1) |
| I used social media applications (WhatsApp, Facebook Messenger…) | 38 (40.9) |
| I called the medical provider’s office | 7 (7.5) |
| I do not communicate the interaction | 4 (4.3) |
| I do not have any referral medical provider | 33 (35.5) |
| Other^d^ | 3 (3.2) |
| **Telehealth used for continuing medical education during COVID-19** |  |
| Yes | 71 (76.3) |
| No | 22 (23.7) |
| **Telehealth sessions’ average minutes spent having direct client(s) contact during COVID-19** |  |
| Less than 30 minutes | 16 (17.2) |
| 30 minutes | 29 (31.2) |
| More than 30 minutes | 48 (51.6) |

LDs, Lebanese Licensed Dietitians; COVID-19, SARS-Cov-2 pandemic

a Mean(SD) = Mean standard deviation

^b^ Respondents were able to select all options that applied.

^c^ A nutrition intervention follows nutrition diagnosis in the nutrition care process and is a purposefully planned action (s) designed with the intent of changing a nutrition-related behavior, risk factor, environmental condition, or aspect of health status to resolve or improve the identified nutrition diagnosis(es) or nutrition problems(s).

^d^ Other detailed answers not provided by survey participants

^⁕^ The total number of participants included in this table is 93, as one participant provided incomplete responses for the variables analyzed.
