# Supplementary material for: Telehealth during and beyond the COVID-19 Pandemic: Evidence from licensed dietitians in an emerging economy
Source: PLoS One. 2026 Feb 6;21(2):e0311330. doi: 10.1371/journal.pone.0311330 (PMC12880700; doi:10.1371/journal.pone.0311330)
Supplement: S3 Table — This table outlines the perceptions of 93 Licensed Dietitians (LDs) in Lebanon regarding key factors influencing the use of telehealth during the COVID-19 pandemic. Reported items include technological challenges, professional and institutional support, patient engagement, and perceived advantages of telehealth for delivering nutrition care. (DOCX) [file pone.0311330.s005.docx]

| **Table 3.** Reported Barriers, Facilitators and Benefits of [telehealth](https://www.sciencedirect.com/topics/nursing-and-health-professions/telehealth) Use during COVID-19 in Lebanon as reported by LDs (N=93)^⁕^ | |
| --- | --- |
| **Telehealth Barriers, Facilitators and Benefits during COVID-19** | **Responsesa** |
| **Barriers experienced by delivering nutrition care via Telehealth^b^** | **N (%)** |
| Providers not having an internet access | 22 (23.7) |
| Bad connection during sessions | 69 (74.2) |
| Discomfort delivering nutrition care via Telehealth | 14 (15.1) |
| Not being able to conduct or evaluate nutrition assessment, monitoring and evaluation | 30 (32.3) |
| Not being able to deliver some routine nutrition interventions via Telehealth | 15 (16.1) |
| Not having remote access to the electronic health record at my home | 13 (14.0) |
| Not having appropriate equipment to deliver Telehealth | 24 (25.8) |
| Difficulty with establishing relationships/therapeutic alliance via Telehealth | 14 (15.1) |
| Lack of patient referrals from medical providers | 22 (23.7) |
| Lack of employer support | 2 (2.2) |
| Patients not interested in having any nutrition services via Telehealth | 23 (24.7) |
| Patients not interested in having any nutrition services at time of COVID-19 | 15 (16.1) |
| Patients preferred face-to-face consultations | 57 (61.3) |
| Patients not having internet access | 36 (38.7) |
| Patients not having landline phones or cellphones | 4 (4.3) |
| Patients not cooperant during Telehealth consultations | 17 (18.3) |
| Patients lack in technical literacy | 31 (33.3) |
| Concerns about privacy and confidentiality | 8 (8.6) |
| Othersc | 49 (52.7) |
| **Telehealth facilitators experienced by delivering nutrition care via Telehealth^b^** | |
| COVID-19 created an atmosphere of technology acceptance and penetration | 65 (69.9) |
| Past experience with Telehealth nutrition care | 11 (11.8) |
| Ease of use | 53 (57.0) |
| Increased communication with patient | 39 (41.9) |
| Increased communication with other health professionals | 28 (30.1) |
| Othersc | 24 (25.8) |
| **Benefits experienced by delivering nutrition care via Telehealth^b^** | |
| Promoting compliance with social distancing measures of COVID-19 | 50 (53.8) |
| Scheduling and time flexibility | 78 (83.9) |
| Decrease costs related to practice (rent, transportation, equipment, stationary...) | 72 (77.4) |

LDs, Lebanese Licensed Dietitians; COVID-19, SARS-Cov-2 pandemic

a Does not include missing responses

^b^ Respondents were able to select all options that applied.

^c^ Other detailed answers not provided by survey participants

^⁕^ The total number of participants included in this table is 93, as one participant provided incomplete responses for the variables analyzed
