# Supplementary figures and images for: Telehealth during and beyond the COVID-19 Pandemic: Evidence from licensed dietitians in an emerging economy
Source: PLoS One. 2026 Feb 6;21(2):e0311330. doi: 10.1371/journal.pone.0311330 (PMC12880700; doi:10.1371/journal.pone.0311330)

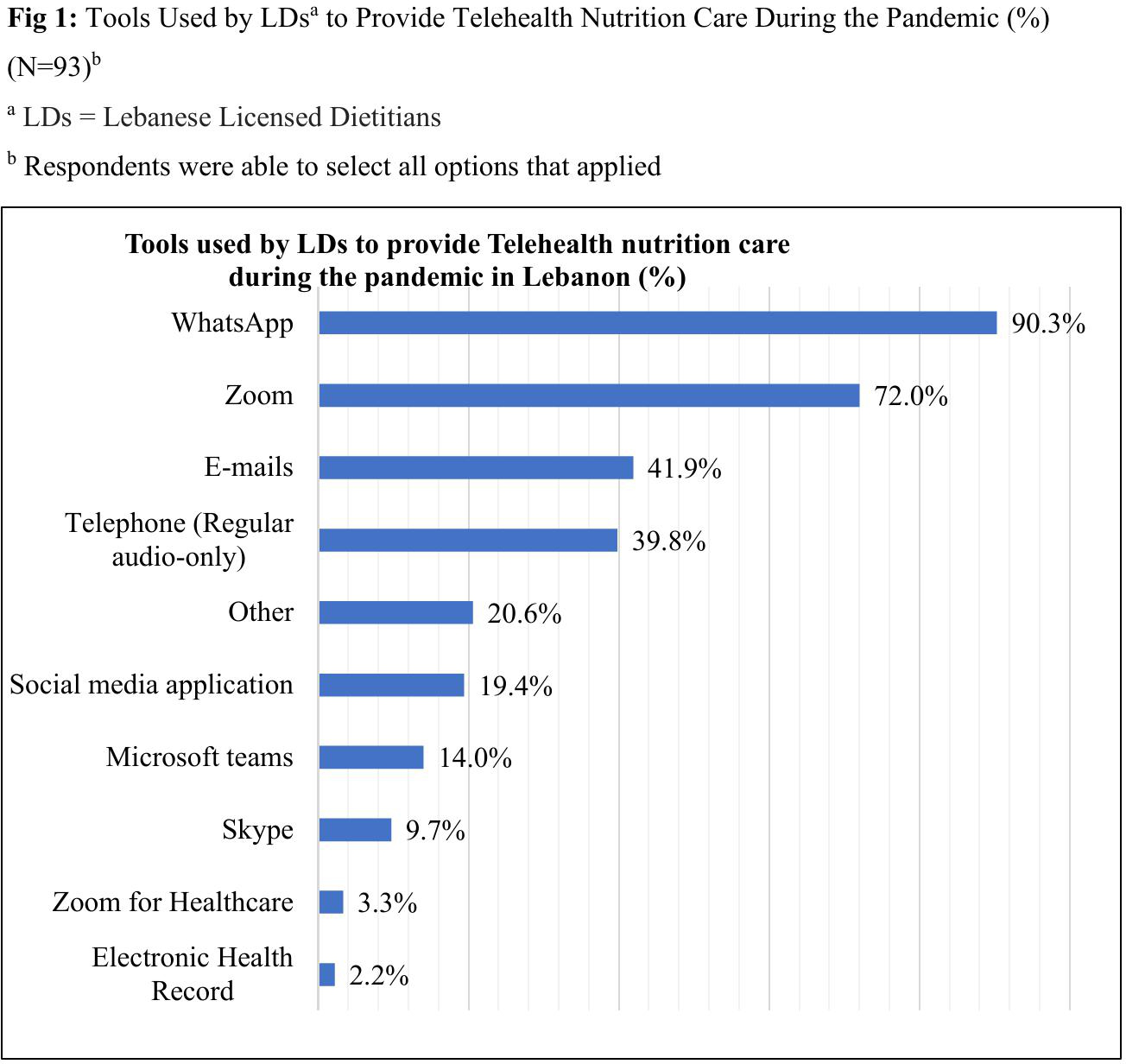

Supplement: S1 Fig — Legend: This figure illustrates the types of tools and platforms utilized by 93 Licensed Dietitians (LDs) in Lebanon to deliver nutrition care via telehealth during the COVID-19 pandemic. Reported tools include videoconferencing applications, phone calls, messaging apps, and social media platforms. WhatsApp (90.3%) and Zoom (72.0%) were the most used platforms, followed by email (41.9%) and regular telephone calls (39.8%). Respondents were allowed to select multiple options. Other tools included social media applications, Microsoft Teams, Skype, Zoom for Healthcare, and electronic health records. Percentage reflects the proportion of respondents who indicated using each tool. (TIF) [file pone.0311330.s006.tif]

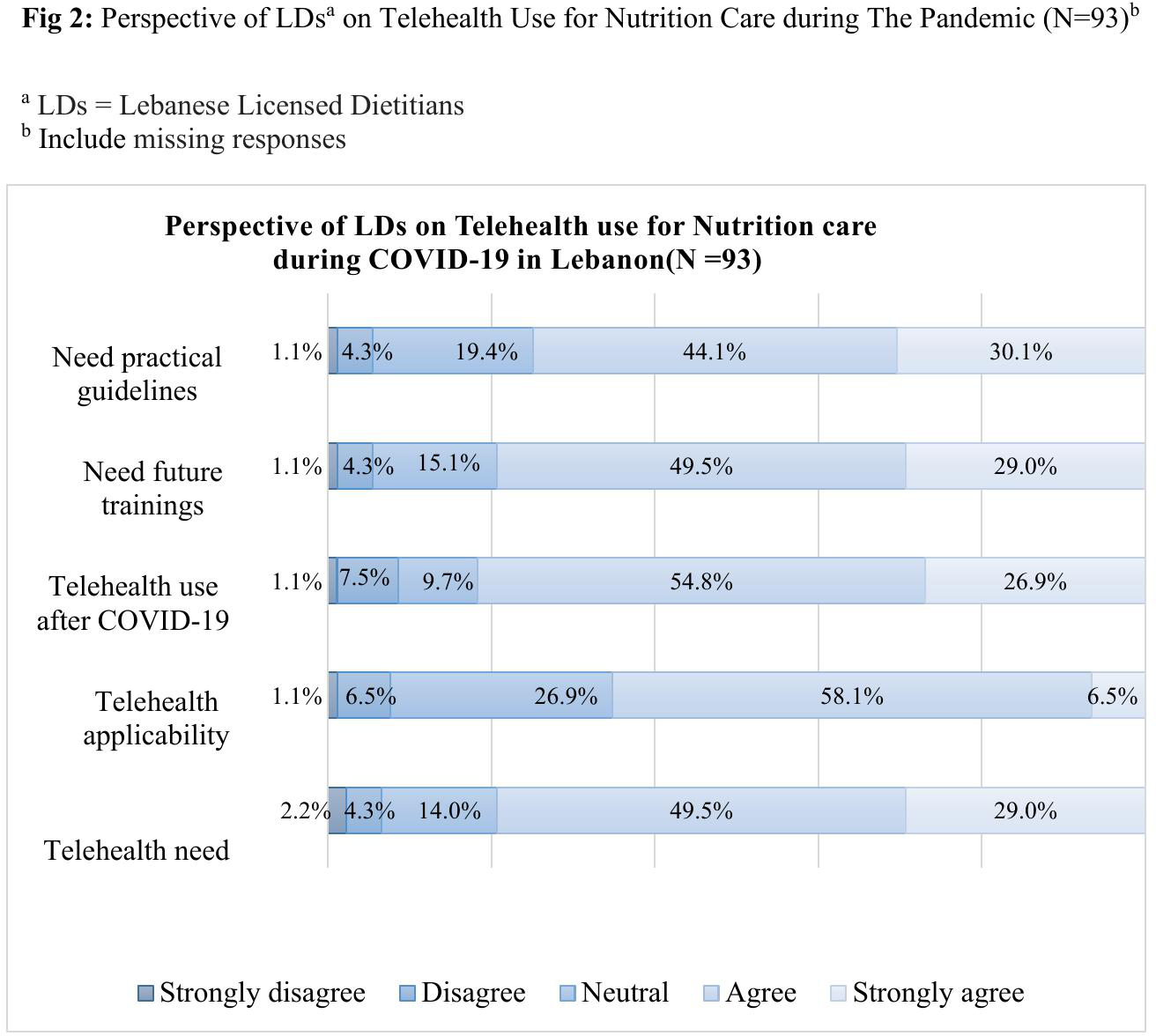

Supplement: S2 Fig — Legend: This figure presents the perceptions of 93 Licensed Dietitians (LDs) regarding telehealth use for nutrition care during the COVID-19 pandemic related to applicability, effectiveness, patient engagement, and professional confidence associated with telehealth delivery. On a Likert Scale, respondents rated their level of agreement with five statements addressing the need for practical guidelines, future training, continued telehealth use post-pandemic, the applicability of telehealth in the Lebanese Context, and the overall need for telehealth in Lebanon. Responses are shown across five categories: strongly disagree, disagree, neutral, agree, and strongly agree. (TIF) [file pone.0311330.s007.tif]

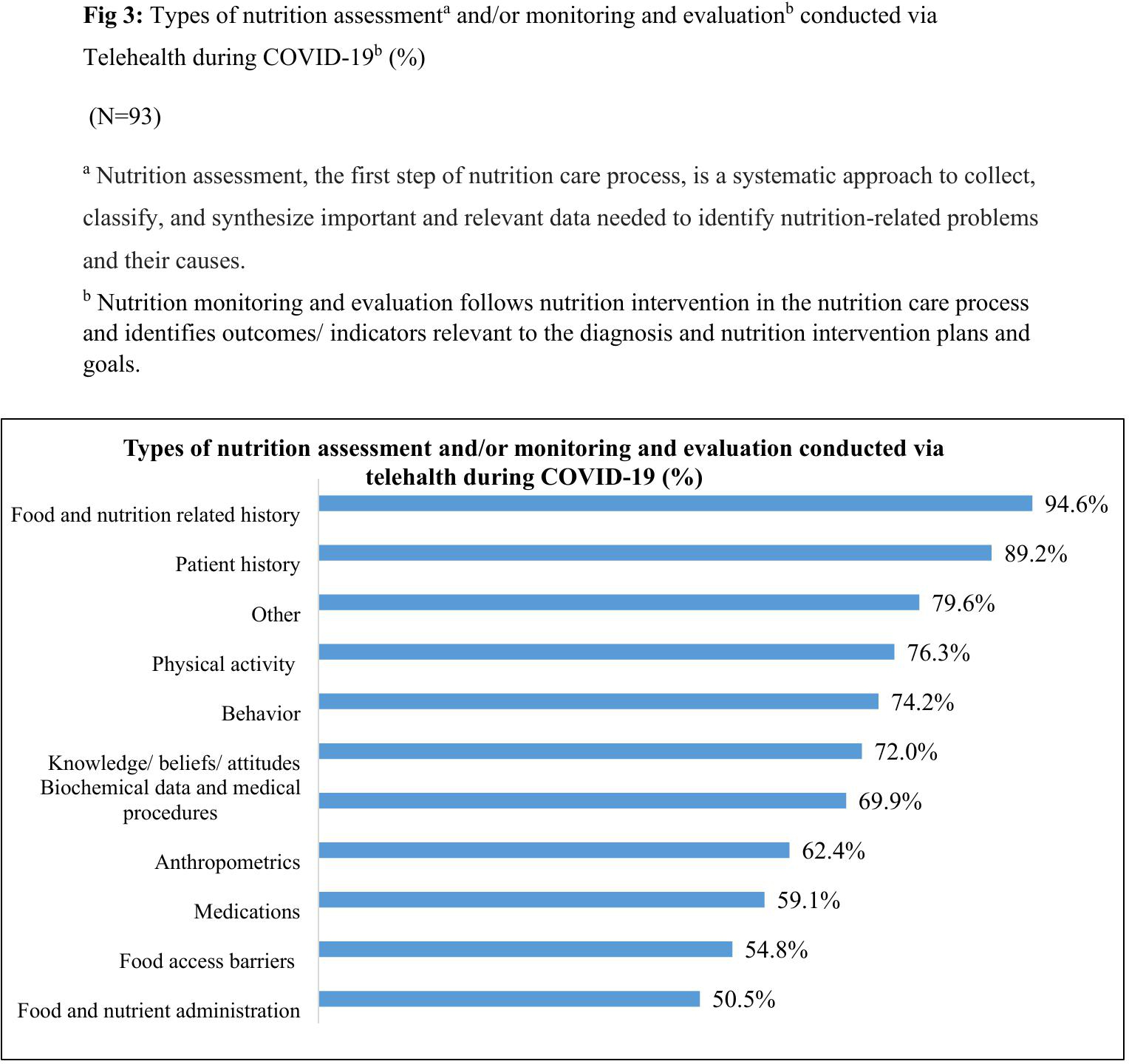

Supplement: S3 Fig — Legend: This figure shows the types of nutrition assessment and monitoring, or evaluation methods performed by 93 Licensed Dietitians (LDs) during the COVID-19 pandemic. Categories include dietary recall, anthropometric tracking, clinical and biochemical data collection, and behavioral assessments. Percentages represent the share of respondents who reported conducting each assessment type remotely. (TIF) [file pone.0311330.s008.tif]
